# C-phycocyanin attenuates RANKL-induced osteoclastogenesis and bone resorption in vitro through inhibiting ROS levels, NFATc1 and NF-κB activation

**DOI:** 10.1038/s41598-020-59363-y

**Published:** 2020-02-13

**Authors:** Mohammed S. AlQranei, Hanan Aljohani, Sunipa Majumdar, Linda T. Senbanjo, Meenakshi A. Chellaiah

**Affiliations:** 1 Department of Oncology and Diagnostic Sciences, School of Dentistry, University of Maryland, Baltimore, MD USA; 2 Preventive Dental Sciences Department, School of Dentistry, Imam Abdulrahman Bin Faisal University, Dammam, Saudi Arabia; 3 Department of Oral Medicine and Diagnostics Sciences, King Saud University, School of Dentistry, Riyadh, Saudi Arabia

**Keywords:** Cell biology, Cell death, Cell signalling, Cytoskeleton

## Abstract

Excessive bone loss occurs in inflammatory disorders such as periodontitis and osteoporosis. The underlying mechanism is related to the differentiation of macrophages into multinucleated giant osteoclasts and their bone resorptive activity. C-Phycocyanin (C-PC) is a phycobiliprotein extracted from the blue-green algae, which has been shown to have various pharmacological effects. The role of C-PC on bone metabolism needs revelation. In this study, we determined the effectiveness of C-PC as an inhibitor of osteoclast differentiation, activity, and survival *in vitro*. We found that C-PC strongly inhibited the differentiation of macrophages to TRAP-positive osteoclasts, distinctive osteoclast specific podosomal organization, and dentine matrix resorption without any cytotoxicity. Also, it suppressed the expression of osteoclast specific markers, such as cathepsin K and integrin β3 at mRNA and protein levels. RANKL mediated signaling utilizes reactive oxygen species (ROS) for the differentiation of osteoclasts. C-PC attenuated RANKL stimulated ROS. Mechanistic studies indicate that C-PC has the potential to reduce osteoclast formation via blocking the degradation of cytosolic IκB-α and hence, the activation of downstream markers such as c-Fos and NFATc1. However, it does not have any effect on osteoblast-mediated bone formation *in vitro*. Collectively, our data suggest that C-PC may be utilized as a therapeutic agent that can target bone loss mediated by excessive osteoclastic bone resorption without affecting osteoblastic activity in bone.

## Introduction

Bone remodeling is a physiologically orchestrated process in which a simultaneous interplay between bone resorption and bone formation facilitates the development and maintenance of the skeletal tissues. However, an abnormal increased or decreased bone resorption rate is involved in the pathophysiology of multiple skeletal disorders^1,2^. Osteolytic diseases, such as osteoporosis and periodontitis, carry a serious health concern. The net outcome of such pathological conditions is the loss of healthy and supportive bone due to the activation of osteoclasts^3,4^.

Osteoclasts are multinucleated-giant cells that are functionally recognized as primary bone-resorbing cells. Osteoclasts express tartrate-resistant acid phosphatase (TRAP), which is considered as one of the biomarkers. Osteoclasts are highly migratory cells, and their migration is dependent on rapid changes in their actin cytoskeletal structures. Osteoclasts do not have the focal adhesion attachment structures typical of other cells^5^. Osteoclasts plated on glass coverslips demonstrated discrete dot-like podosome structures at the periphery, and, in some cells, podosomes were observed as ring-like structures^6,7^. These types of podosomal or actin-ring like structures represent the unique characteristics that can demarcate the mature osteoclasts from osteoclast precursors. Osteoclasts are highly migrated cells and dependent on podosomes for their cellular motility^6,8,9^.

The central regulators of osteoclasts differentiation are two cytokines, namely receptor activator of nuclear factor kappa B ligand (RANKL) and macrophage colony-stimulating factor (M-CSF)^10,11^. M-CSF is a prerequisite for the proliferation and survival of monocytic lineage cells. It also promotes the differentiation of bone marrow precursors to osteoclast precursors and increases the expression of RANK in bone marrow cells^10,12^. RANKL, which is also called the osteoclast differentiation factor (ODF), is the essential cytokine needed for the induction of mononuclear cell fusion to form the multinucleated osteoclasts^10,13^. Upon binding of RANKL to RANK, a series of downstream signaling is activated, including the activation of the NF-κB pathway. This activation invokes the stimulation of c-Fos, which consequently activates the nuclear factor of activated T cells cytoplasmic 1 (NFATc1), a master regulator of osteoclastogenesis^11,14,15^. Therefore, targeting such a signaling pathway may restrain the differentiation of osteoclasts, which could present an effective treatment strategy for osteolytic inflammatory diseases.

C-Phycocyanin (C-PC) is a phycobiliprotein found primarily in the blue-green algae such as *Spirulina platensis*. This water-soluble protein pigment has been often used as a nutritional dietary supplement in many countries^16,17^. It exhibited several pharmacological effects, including reactive oxygen species (ROS)-scavenging actions, anti-oxidant^18^, hepatoprotective^17^, and anti-arthritic^19^. Furthermore, C-PC has shown anti-inflammatory effects in several experimental models, *in vitro* and *in vivo*^19–22^. Inhibition of cyclooxygenase-2 activity has been reported as one of the significant anti-inflammatory effects of C-PC^23^. Later, C-PC has demonstrated anti-inflammatory effects on lipopolysaccharide-stimulated RAW 264.7 macrophages. It interferes with the degradation of the cytosolic IκB-α after lipopolysaccharide stimulation, which consequently suppresses the activation of the NF-κB pathway^24^. However, the C-PC effect on osteoclast differentiation and resorption activity is not well understood and needs elucidation.

In this paper, we report the potential inhibitory effect of C-PC on the formation of osteoclasts from RAW 264.7 (henceforth denoted as RAW cells) murine macrophages. C-PC decreased osteoclastogenesis in RANKL-stimulated RAW cells by blocking the degradation of cytosolic IκB-α and hence, the activation of downstream markers such as c-Fos and NFATc1. C-PC also attenuates RANKL-mediated intracellular ROS generation. However, it does not affect the differentiation or function of osteoblasts. These data suggest an anti-osteoclastogenic role of C-PC that can be utilized to prevent inflammatory bone loss.

## Results

### C-Phycocyanin (C-PC) inhibits RANKL-induced differentiation of osteoclasts from RAW 264.7 cells

Osteoclasts are the only bone cells resorb bone. RANKL-RANK-mediated signaling mechanisms are essential for osteoclast differentiation^10^. First, we evaluated the ability of C-Phycocyanin (C-PC) to inhibit osteoclast differentiation from RAW cells. The cytotoxicity was assessed by MTT assay in cells treated with varying concentrations of C-PC (Supplementary Fig. S1). Results demonstrated a non-toxic nature of C-PC up to a concentration of 100 µg/ml of medium, and significant cytotoxicity was observed at the concentration of 150 µg/ml. Therefore, we used C-PC at the concentration of 25 or 50 µg/ml in the results shown below.

**Figure 1:**
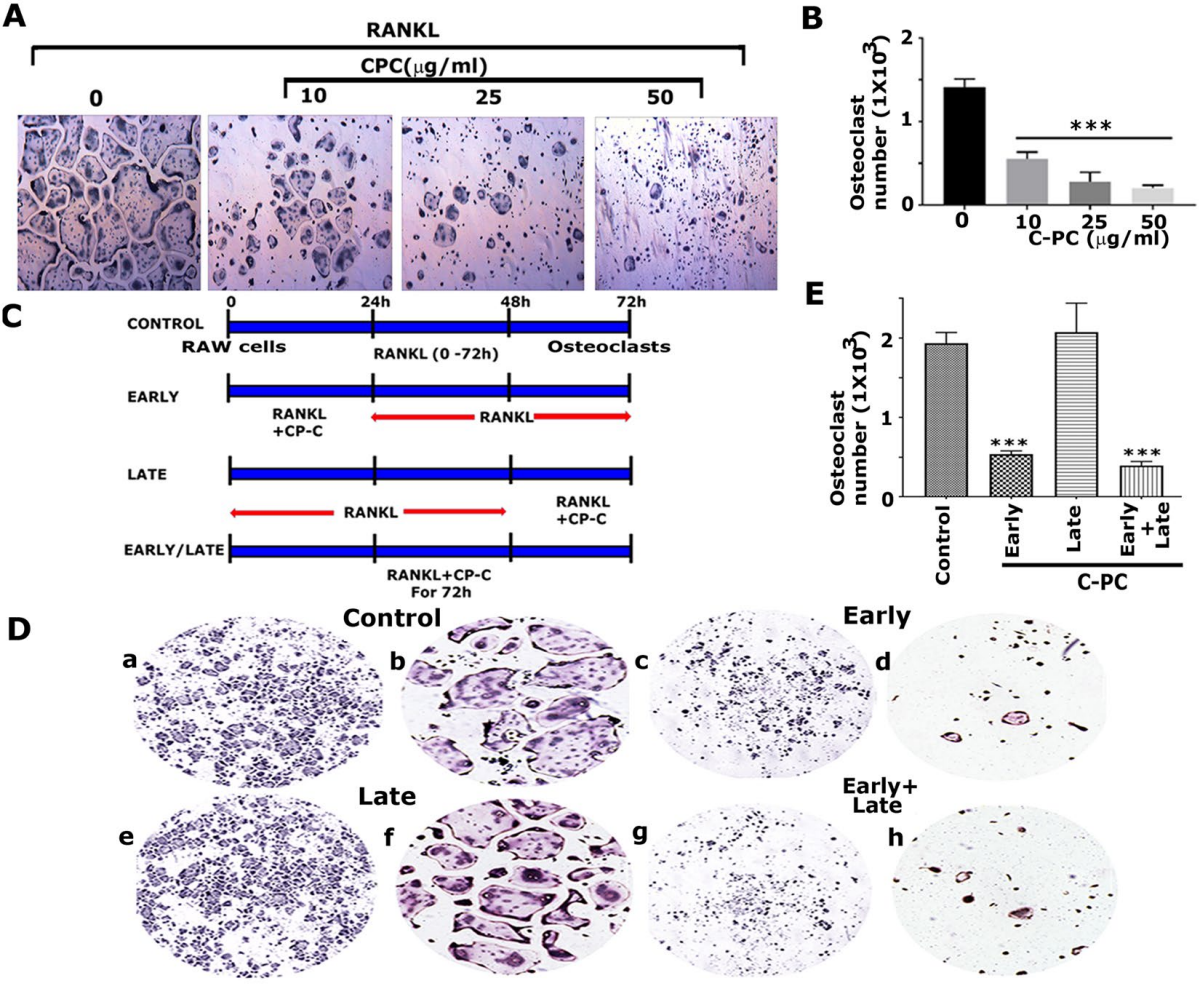
Analysis of the effect of C-PC on RANKL-mediated osteoclast differentiation. (**A**) Effect of different doses of C-PC (10, 25, and 50 µg) on osteoclast differentiation. Phase-contrast microscopy images of TRAP stained osteoclasts are shown at indicated doses. Magnification is 4X. **(B)** The number of TRAP +ve multinucleated osteoclasts were counted in osteoclasts untreated (0) or treated with 10, 25, and 50µg C-PC for 72 h. Statistical analysis was performed to determine the dose-dependent inhibitory effect of C-PC on osteoclasts differentiation as compared with control untreated (0) cells. **(C)** Identification of the time-dependent effect of RANKL and C-PC on the osteoclast differentiation. The diagrammatic sketch demonstrates the treatment strategy of RAW cells with RANKL and C-PC (25 µg/ml). **(D)** Representative images of TRAP stained osteoclasts in response to the treatment strategy shown in panel C.TRAP stained osteoclasts in panels a,c,e, and g were taken with 4X objective (magnification: X40), while panels in b,d,f, and h were taken with 10X objective (magnification: X100). **(E)** The number of TRAP +ve multinucleated osteoclasts were counted in all treatment groups. Statistical analysis was performed to compare the number in the treatment groups to the control group. One-way ANOVA was applied, and values were expressed as mean ± SD. ***P < 0.001.

The enzyme tartrate-resistant acid phosphatase (TRAP) is expressed by osteoclasts and has been used as a histochemical marker for mature osteoclast and their precursors. In our studies, we used the TRAP staining method to demonstrate the effect of different doses of C-PC (10, 25, and 50 µg/ml) on osteoclast differentiation. Hence, RAW cells treated with both RANKL and different doses of C-PC for 72 hours were subjected to TRAP staining (Fig. 1A). RAW cells treated with RANKL alone were used as controls (Fig. 1A; 0 µg/ml). C-PC decreased the number of TRAP-positive multinucleated osteoclasts in a dose-dependent manner (Fig. 1A, 10–50 µg/ml). Since mononuclear macrophages also express TRAP enzyme^25^, undifferentiated RAW cells were also stained in C-PC treated cells at all doses. TRAP-positive mature multinucleated osteoclasts were counted and provided in a graph (Fig. 1B). The highest decrease of osteoclast number was found at 50 µg/ml of C-PC (Fig. 1A,B).

To further define the stage at which C-PC inhibits osteoclastogenesis, RAW cells were treated with C-PC, as shown in the diagrammatic sketch (Fig. 1C) and as described in the methods section. After the treatments, as indicated in Fig. 1C, cells were stained for TRAP enzyme to determine the inhibitory effect of C-PC on osteoclast differentiation *in vitro*. Cells were photographed in a Cytation 5 cell imaging multimodel plate reader under 4X (Fig. 1D- panels a, c, e, and g) and 10X (Fig. 1D- panels b, d, f, and h) objectives. We show here that the number of TRAP-positive osteoclasts was significantly reduced in the early (Fig. 1D, panels c and d) and early + late groups (panels g and h). However, the addition of C-PC after 48 h of RANKL treatment (late group) had no significant inhibitory effect on osteoclast formation (Fig. 1D, panels e and f). The number of TRAP-positive osteoclasts in the late group is comparable to the control group (Fig. 1D, panels a and b). A quantitative analysis of the number of TRAP ^+ve^ osteoclasts is provided in Fig. 1E. Mature TRAP ^+ve^ osteoclasts were counted in ~4 to 5 fields/treatment from three different experiments and provided as a graph (n = 3) (Fig. 1E). These results demonstrate that C-PC inhibits the early stage of differentiation of osteoclasts from RAW cells. Therefore, we used the strategy of early-stage inhibition (0–24 h) in the studies shown below.

### Immunoblotting analysis of apoptosis and osteoclast markers in RAW cells treated with C-PC

**Figure 2:**
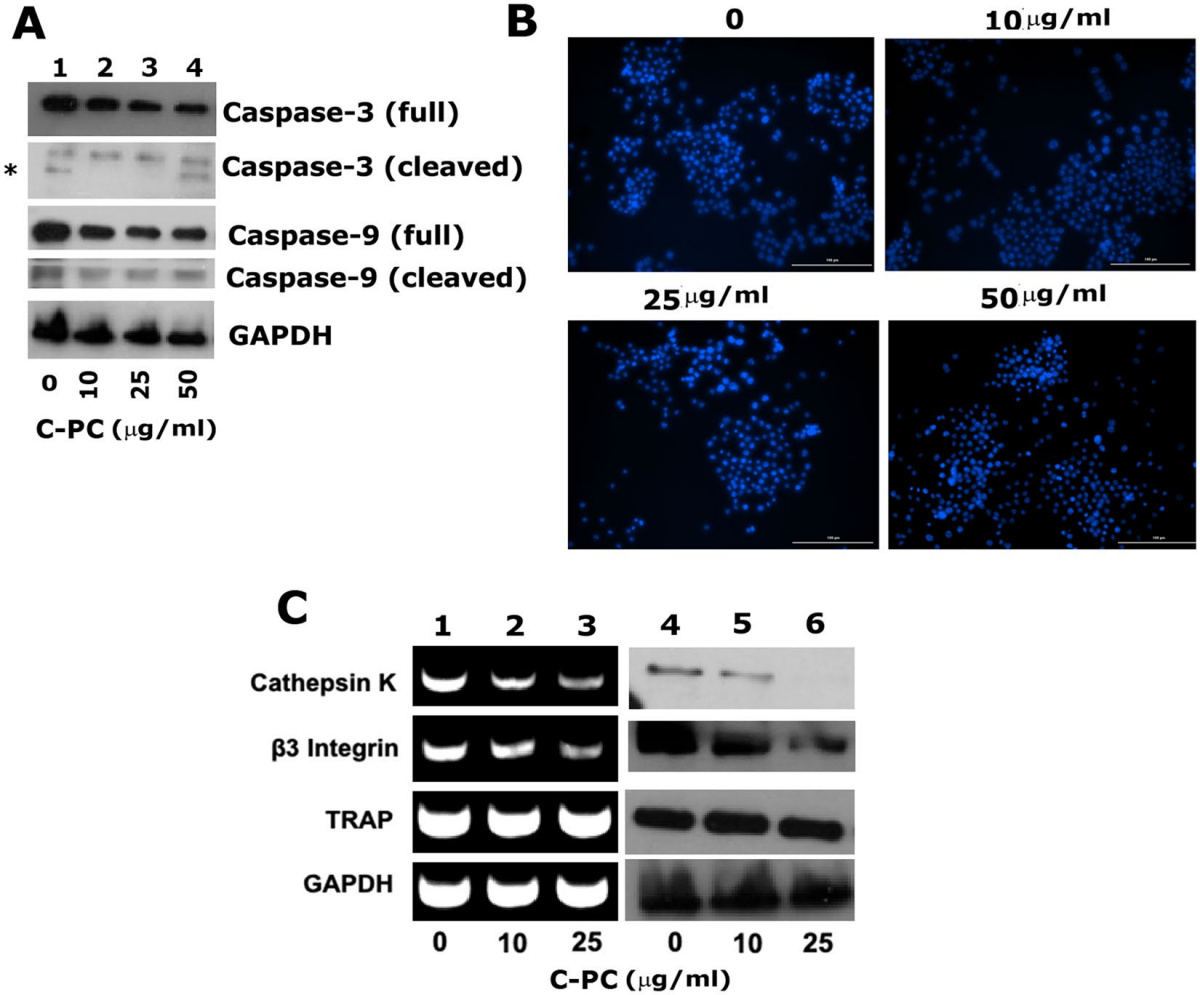
Analysis of the effect of C-PC on the expression of apoptotic markers and osteoclast-specific markers. (**A**) Immunoblotting analyses with antibodies to full caspase-3 (**~**35 kDa), cleaved caspase-3 (**~**19 kDa), full caspase-9 (**~**49 kDa), cleaved caspase-9 (**~**39 kDa), and GAPDH (loading control; **~**37 kDa) are shown. *Indicates non-specific band in lane 1 and 4. **(B)** Evaluation of apoptosis by DAPI (4′,6′-diamidino-2-phenylindole) staining in osteoclasts untreated or treated with C-PC in the presence of RANKL. Representative phase-contrast micrographs are shown. Magnification is X100. **(C)** Analysis of the effect of C-PC on the expression of osteoclast-specific markers at the mRNA level by RT-PCR analysis and at the protein level by immunoblotting analysis with indicated antibody. RT-PCR analyses for Cathepsin K (400 bp), integrin β3 (305 bp), TRAP (220 bp), and GAPDH (loading control; 324 bp) are shown in (lanes 1–3). A parallel batch of cell culture was used for the lysate preparation and immunoblotting analyses (lanes 4–6) with antibodies to cathepsin K (**~**29 kDa), integrin β3 (**~**97 kDa), TRAP (**~**42 kDa), and GAPDH (loading control; **~**37 kDa). Equal loading was assessed by sequential blotting of the same membrane with cathepsin K and TRAP antibody after stripping. Similarly, equal loading was assessed in β3 integrin blot by sequential immunoblotting with a GAPDH antibody after stripping. Results shown represent one of the three experiments performed with similar results. Scanned uncropped autoradiograms are presented in Supplementary Figs. S3–S5. The rectangle in each image represents the corresponding protein(s) shown in the immunoblotting analysis of Fig. 2.

We then performed immunoblotting analysis with a caspase −3 and −9 antibody to identify whether the decrease in osteoclast number by C-PC treatment is due to apoptosis. Immunoblotting analysis demonstrated no changes in the levels of inactive pro-enzymes known as uncleaved caspases, which are indicated as full in Fig. 2A. Similarly, no changes in the levels of cleaved caspases (−3 and −9) were observed, and also minimal levels of these proteins were observed in untreated, and C-PC treated osteoclasts (Fig. 2A). C-PC did not have any effect on the cleavage of either caspase-3 or caspase-9. Minimal levels of cleaved enzymes indicate that apoptotic pathways are not stimulated. This is corroborated in RAW cells stained with 4′,6′-diamidino-2-phenylindole (DAPI) stain (Fig. 2B, 10–50 μg). Although DAPI staining is not the primary marker to evaluate apoptosis, we used this method to determine the nuclear morphology. No significant changes in the nuclear morphology were observed in C-PC untreated and treated cells. No changes in caspase (−3 and −9) levels suggest that the decrease in the number of mature osteoclasts in C-PC treated RAW cells is not due to the activation of apoptosis-related mechanisms.

Active osteoclasts are characterized by the expression of TRAP, Cathepsin K, and integrin αvβ3. These are expressed in RANKL dependent manner^26^. Integrin-mediated signaling is required for osteoclast adhesion^27^. Therefore, to further validate the inhibitory effect of C-PC, we analyzed the expression levels of specific molecular markers (Cathepsin K, integrin β3, and TRAP) of osteoclast differentiation at mRNA and protein levels (Fig. 2C, lanes 1–6). Expression levels of cathepsin K and integrin β3 decreased at mRNA levels in a dose-dependent manner in C-PC treated osteoclasts (Fig. 2C, lanes 1–3). This corresponds with a decrease in the protein levels of respective proteins (Fig. 2C, lanes 5 and 6). No significant changes in the expression TRAP was observed at mRNA and protein levels in untreated (Fig. 2C, lanes 1 and 4) or C-PC treated cells (lanes 2, 3, 5, and 6). These results suggest that integrin signaling plays a crucial role in RANKL-induced osteoclast differentiation.

### Analysis of the distribution of podosomes and resorption activity in raw cells treated with C-PC

**Figure 3:**
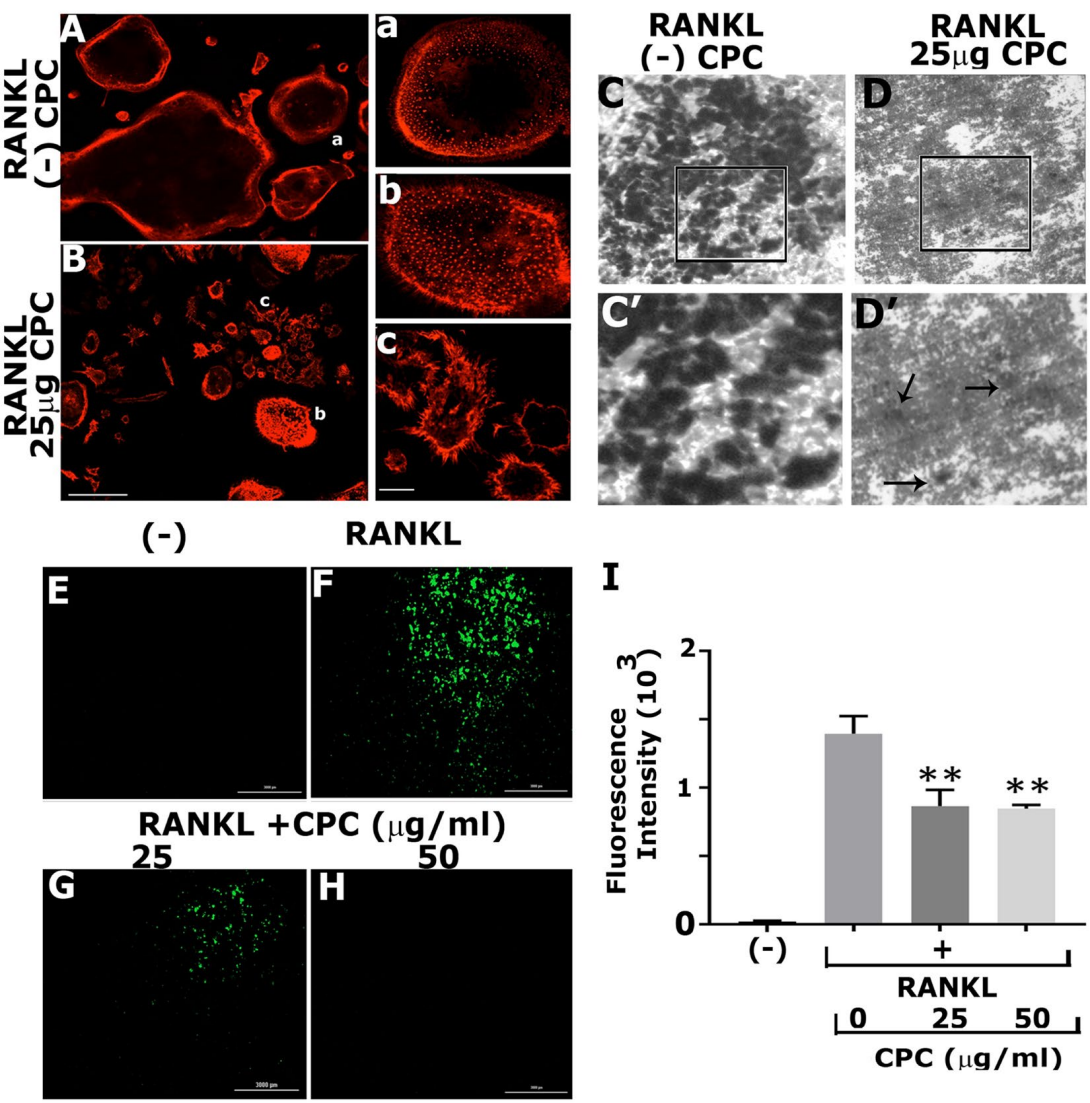
Analysis of the effect of C-PC on osteoclast-specific phenotype, function, and markers. (**A**,**B)** Actin staining of osteoclasts with rhodamine-phalloidine: RAW cells were seeded on coverslips and treated with and without C-PC (50 μg) in the presence of RANKL. After 72 hours, cells in both groups were fixed and stained for rhodamine-phalloidine. Pictures on the right side (a–c) represent magnified areas indicated in (**A**,**B)**. **(C**,**D)** Analysis of the resorption activity in osteoclasts treated with and without C-PC (50 μg) in the presence of RANKL. Representative phase-contrast micrographs are shown. The rectangle in panels (C,D) represents the area of the image, which is magnified in C’ and D’. Magnifications are X100 (**C**,**D**) and X400 (C’ and D’). Arrows in D’ point to shallow pits. (**E**–**I**) Evaluate the effect of C-PC on the changes in reactive oxygen species (ROS) levels using dichlorofluorescein diacetate (DCFDA) assay method. Representative images of ROS (+) cells during RANKL-induced osteoclastogenesis are shown. Varying doses of C-PC (25, 50) μg reduces the green fluorescence intensity. Staining represents the extent of ROS generation. (**I**) Quantification of the number of ROS (+) cells in all groups is provided in the graph. C-PC reduced ROS levels induced by RANKL. One-way ANOVA was applied, and values were expressed as mean ± SD. **P < 0.01.

Integrin αvβ3 is critical for osteoclast adhesion and migration, and αvβ3 signaling regulates the formation of podosomes in osteoclasts^28^. Furthermore, the transformation from mononuclear cells into a multinucleated osteoclast is noticeable cytoskeletally by the formation of podosomes, which is also essential for osteoclast migration and bone resorption^29^. The organization of actin filaments was determined by staining of osteoclasts with rhodamine-phalloidin (Fig. 3). Raw cells not treated with C-PC but treated with RANKL differentiated into mature osteoclasts with peripheral belts of podosomes. The presence of belt-like podosomes at the periphery is a sign of fully mature osteoclasts (Fig. 3, panels A and a). We have shown here the presence of TRAP stained RAW cells at doses of 25 and 50 μg of C-PC (Fig. 1A). Actin staining also demonstrated the presence of numerous round, flattened, and elongated RAW cells (Fig. 3, panels B, b, and c). Actin distribution was observed in the plasma membrane of these cells (Fig. 3B). Magnified images of round and elongated RAW cells are shown in Fig. 3 (panels b and c). As shown by others in primary human macrophages^30^, some RAW cells demonstrated actin-rich podosomes throughout the cells (Fig. 3, panel b). Flattened elongated cells demonstrated filopodia-like extensions from the periphery of the membrane (Fig. 3, panel c).

Furthermore, C-PC reduced the resorption activity significantly in osteoclasts plated on dentine slices (Fig. 3D,D’), and only a few shallow pits were observed on the dentine matrix (Fig. 3D’, indicated by arrows). However, C-PC untreated cells (−) demonstrated multiple overlapping resorption pits (Fig. 3C,C’). These observations suggest that C-PC attenuates the differentiation of RAW cells into mature osteoclasts, and C-PC treated cells have features of macrophages, which failed to resorb the dentine matrix.

### C-PC attenuates the generation of RANKL-induced ROS

From the results shown in Fig. 2, we identified that apoptosis is not a mechanism for the reduced differentiation of osteoclasts in C-PC treated cells (Fig. 2). To further evaluate the effect of C-PC on osteoclastogenesis, we semi-quantitatively measured the changes in reactive oxygen species (ROS) levels using dichlorofluorescein diacetate (DCFDA) assay method (Fig. 3E–H). We show here that the fluorescence intensity observed in osteoclasts generated with RANKL (Fig. 3, panel F) is significantly reduced in cells treated with C-PC in a dose-dependent manner (Fig. 3, panels G and H). The bar graph shows the quantification of relative fluorescence intensity in RANKL only, and RANKL + C-PC treated cells (25 and 50 μg) from three different experiments (Fig. 3, panel I). RAW cells not stimulated with any treatment were used as a negative control to detect the basal level of ROS (Fig. 3E; (−) in Fig. 3I). ROS components are essential in the regulation of differentiation of osteoclasts. ROS produced at more than one subcellular site of macrophages was shown to regulate osteoclast differentiation^31^. Our results show that reactive oxygen species have a role in osteoclastogenesis. Future studies will focus on the subcellular target site of C-PC in RAW cells.

### C-PC suppressed the activation of NFATc1 and c-Fos

**Figure 4:**
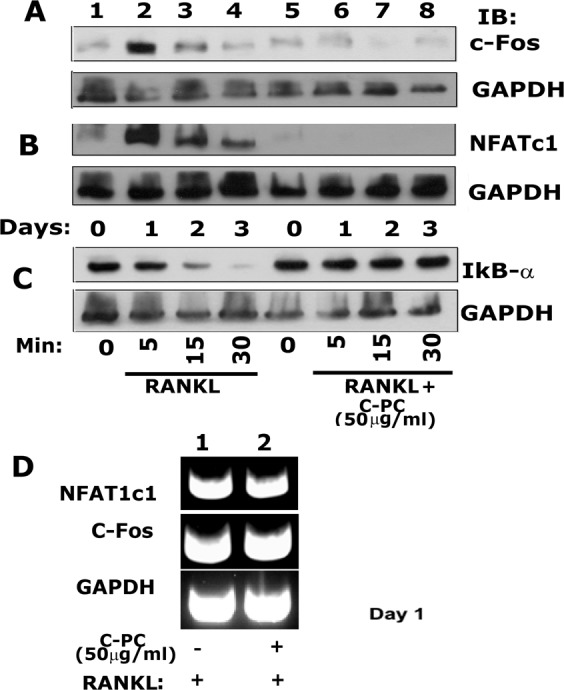
Immunoblotting analysis of the effect of C-PC on the expression levels of c-Fos (**A**), NFATc1 (**B**), and IκB-α (**C**). (**A**–**C**) An equal amount of lysate proteins were used for immunoblotting analyses with antibodies to c-Fos (~62 kDa), NFATc1 (~90 kDa), and IκB-α (**~**39 kDa) to detect the expression at protein levels. **(D)** RT-PCR analyses were done to evaluate the C-PC effect on the expression of NFATc1 (350 bp) and c-Fos (350 bp) at the mRNA level. Analyses were done in osteoclasts treated with P-CP for 1day. Each experiment was repeated three times and obtained comparable results. Scanned uncropped autoradiograms are presented in Supplementary Figs. S6–S9. Corresponding immunoblots are shown in (**A**–**C**).

In order to identify the underlying mechanism for the inhibitory effect of C-PC on the differentiation of osteoclasts, we investigated the expression levels of NFATc1, c-Fos, and IκB-α. NFATc1 was shown as a master regulator of osteoclastogenesis. The activation of NFATc1 by RANKL occurs via the signaling pathway mediated by the NF-κB and c-Fos^32^. Accordingly, RAW cells were treated with RANKL or RANKL + C-PC (50 μg/ml) for 0, 1, 2, and 3 days. Immunoblotting analysis was performed to determine the protein levels of c-Fos (Fig. 4A), NFATc1 (Fig. 4B), and IκB-α. (Fig. 4C). The expression of c-Fos and NFATc1 was more on day 1 and then decreased gradually on days 2 and 3 in RAW cells treated with RANKL (Fig. 4A,B; lanes 2–4). C-PC reduced significantly the RANKL-induced expression levels of NFATc1 and c-Fos (Fig. 4A,B, lanes 6–8) below the basal levels observed in RAW cells not treated with RANKL (Fig. 4A,B; lane 1). Treatment of cells with 50 μg/ml C-PC demonstrated a compelling decrease in the expression of both NFATc1 and c-Fos at indicated time points. The decrease was maximal at day1 (lanes 6 in A and B) as compared with the corresponding C-PC untreated control (lane 2 in A and B).

We then determined the expression levels of NFATc1 and c-Fos on day 1 by RT-PCR analysis. We chose day 1 because a significant increase in NFATc1 and c-Fos was observed at this time point in response to RANKL (Fig. 4A,B; lane 2), and that increase was significantly reduced by C-PC treatment (Fig. 4A,B, lane 6). There were no changes in the levels of mRNA of NFATc1 and c-Fos was observed in both groups (Fig. 4D), which indicates that the expressions are not transcriptionally regulated.

Others showed that C-PC attenuated lipopolysaccharide-induced NF-κB activation by preventing cytosolic degradation of IκBα^24^. Therefore, we tested whether a similar mechanism exists in RAW cells treated with RANKL and whether C-PC is capable of inhibiting RANKL-induced IκB-α degradation. Since the level of IκBα was very low and not seen on day 1 in our preliminary studies, we used lower time points (5–30 min) to determine the expression levels of IκB-α (Fig. 4C). We observed a time-dependent decrease in IκBα expression in RANKL alone treated cells, which indicates a gradual degradation of IκB-α (Fig. 4C, lanes 2–4). No degradation of IκB-α was detected in all-time points tested for the C-PC/RANKL treated groups. The level of IκB-α was equal to the basal level observed in RANKL and RANKL/C-PC untreated cells at 0 min (Fig. 4C, lanes 1 and 5). Taken together, these results suggest that C-PC inhibits RANKL-mediated osteoclastogenesis by blocking the NF-κB signaling and, hence, attenuate the expression of the downstream proteins NFATc1 and c-Fos at the early stage of osteoclast differentiation.

### C-PC enhanced apoptosis of mature osteoclasts

**Figure 5:**
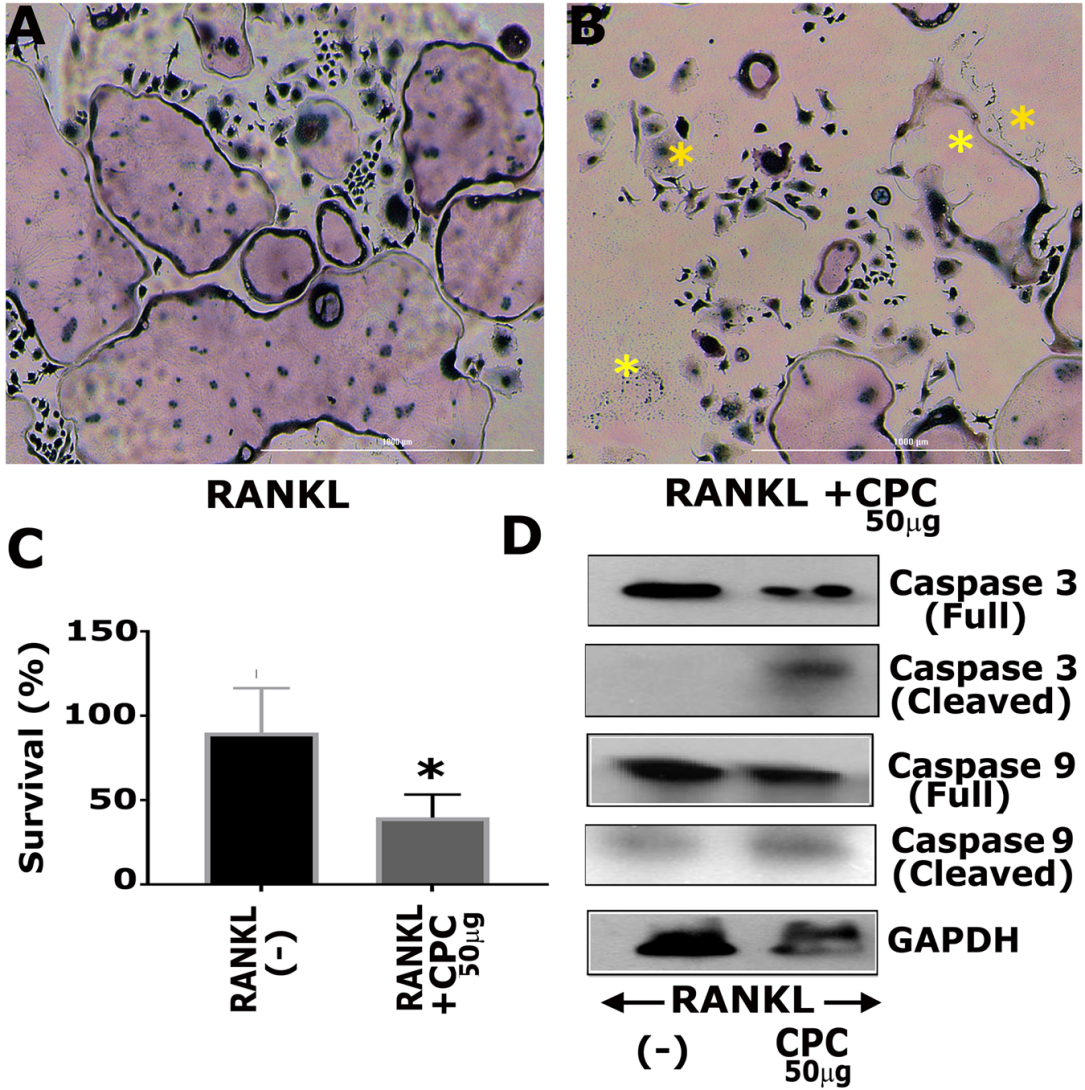
Apoptotic effects of C-PC on mature osteoclasts. Terminally- differentiated osteoclasts were treated with RANKL **(A)** or RANKL + 50 μg C-PC for 12 hours. **(B)** Cells were then fixed and stained for TRAP. Asterisks indicate osteoclasts that are undergoing apoptosis. **(C)** TRAP-stained osteoclasts (survived) were quantitated, and statistical analysis was performed. *P < 0.05, t-test was applied. **(D)** Immunoblotting analyses were done with indicated antibodies to determine the protein levels of apoptotic markers: full caspase-3 (**~**35 kDa), cleaved caspase-3 (**~**19 kDa), full caspase-9 (**~**49 kDa), and cleaved caspase-9 (**~**39 kDa). Equal loading was assessed by immunoblotting with a GAPDH (**~**37 kDa) antibody. Scanned uncropped autoradiograms are presented in Supplementary Fig. S10. The rectangle in each image represents the corresponding protein(s) shown in the immunoblotting analysis of (Panel D).

We have shown in Fig. 2, that there are no changes in the levels of caspases-3 and -9 in cells treated with C-PC at the early stage of differentiation. To determine whether a similar effect occurs in the later stage, mature osteoclasts were cultured in the absence or presence of 50 μg/ml C-PC for 12 hours. Then, osteoclasts were stained for TRAP, and a representative micrograph of untreated (Fig. 5A) and C-PC treated (Fig. 5B) osteoclasts is shown. C-PC treated mature osteoclasts demonstrated reduced viability (Fig. 5B) as compared with untreated osteoclasts (Fig. 5A). The percent survival is decreased considerably in C-PC treated osteoclasts (Fig. 5C). Immunoblotting analysis revealed that a decrease in the survival might be due to the activation of caspase-3 and, to a lesser extent, by caspase-9 in response to C-PC (Fig. 5D). The characteristic morphological changes in osteoclasts (dying osteoclasts) and the activation of caspase-3 suggest that C-PC induces apoptosis in mature osteoclasts and not at the early stage of differentiation (Figs. 1 and 2).

### C-PC did not affect the differentiation and function of osteoblasts

**Figure 6:**
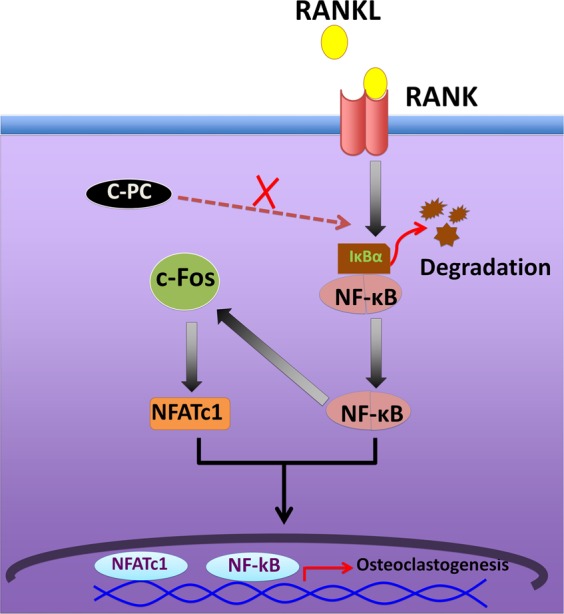
Schematic illustration of the C-PC inhibitory mechanism on RANKL-induced osteoclast formation. NF-κB can act as an upstream factor and mediate RANKL-induced c-Fos and NFATc1 expression. C-PC significantly reduces RANKL-induced NF-κB activation, which therefore reflects not only on the induction of c-Fos and NFATc1 but also on osteoclastogenesis.

Bone remodeling relies on the coordinated action between osteoblasts and osteoclasts^1^. We sought to determine whether C-PC has any effect on alkaline phosphatase (ALP) activity and mineralization processes mediated by mature osteoblasts. Mineralization was assessed by Alizarin Red staining (Supplementary Fig. S2). A rat osteosarcoma cell line (UMR 106 cells) was used, and cells were grown in osteogenic medium (OM) containing ascorbic acid, and β-glycerophosphate for 7 days. Some cultures were treated with 50 μg/ml of C-PC for 7 days in the presence of OM (OM/C-PC). UMR cells grown in the medium with no osteogenic factors failed to demonstrate the formation of minerals and hence did not stain in red by Alizarin Red staining ((−) in Supplementary Fig. S2). Cells grown in OM or OM/C-PC did not show any significant differences in the activity of the ALP or mineralization process (A and B in Supplementary Fig. S2). Taken together, our observations suggest that C-PC may reduce NF-κB signaling and, therefore, reducing the levels of NFATc1 and c-Fos, which resulted in an anti-clastogenic effect. C-PC had no effect on osteoblast differentiation or activity (Fig. 6).

## Discussion

Bone resorption is a significant consequence of inflammatory osteolytic diseases. The increased differentiation and activity of the resorption-competent osteoclasts represent the essential mechanism underlying osteolysis^33^. Osteoclast differentiation is regulated by RANKL/RANK signaling^10^. In the present study, our data showed that C-PC efficiently suppressed RANKL- induced osteoclastogenesis *in vitro*. To our knowledge, this is the first study to explore the effect of C-PC on bone metabolism. At the molecular level, C-PC significantly suppresses multiple pathways downstream to RANK, including NF-κB, ROS, NFATc1, and c-Fos.

In this study, the potential anti-osteoclastogenic effect of C-PC was explored on the murine macrophages cell line, RAW 264.7 cells. RANKL and M-CSF are the primary cytokines responsible for osteoclastogenesis^11^. After exposure to these cytokines, osteoclasts are differentiated following a multi-step process, including a proliferation of the hematopoietic-derived mononuclear precursors, TRAP expression, and fusion of cells. The TRAP^+ve^ multinucleated cells are capable of inducing bone resorption^34^. Based on these theories, a dose-dependent decrease in the number of TRAP^+ve^ osteoclasts suggests a direct inhibitory effect of C-PC on the RANKL-induced osteoclastogenesis. This assay is considered the primary tool to investigate whether a non-cytotoxic compound has an anti-osteoclastogenic activity or not^35–37^.

Moreover, we tested the effect of C-PC during several time points throughout the differentiation process. Our results showed that there is no difference between the (early) and (early + late groups) in terms of a decrease in the number of osteoclasts. However, the (late) group was comparable to the control, which suggests that C-PC may work exclusively in the early phases of osteoclastogenesis.

Podosomes are small adhesion structures that mainly found in highly motile cells^38^. Mature osteoclasts are distinctly characterized by the presence of a well-defined actin belt formed by podosomal organization^39,40^. In this study, we demonstrated the ability of C-PC to inhibit the formation of mature osteoclasts and hence, the formation of the peripheral belt of these podosomal structures. The presence of small diffuse podosomes structures instead of a well-defined peripheral belt illustrates an abundance of macrophages or mononuclear precursors^41^ in C-PC treated cells. These cells failed to form mature resorption-competent osteoclasts.

Moreover, we tested the ability of C-PC to attenuate the resorptive activity of mature osteoclasts by seeding osteoclasts on the dentin chips. RANKL treated cells demonstrated well-demarcated trails of the resorbed area, indicating the process of resorption with cell motility. Cell motility is due to the formation of podosomes. However, C-PC treated osteoclasts failed to exhibit any evidence of resorption or motility. It is also known that the extent of bone resorption is strongly linked with the survival rate of osteoclasts. C-PC accelerated the apoptosis of mature osteoclasts by activation of caspase-3. From these results, it is clear that C-PC inhibits the differentiation, survival, and function of mature osteoclasts.

ROS was generated during cellular stresses, and some of the ROS products include hydrogen peroxide and superoxide radicals^42^. The exposure to RANKL can markedly elevate ROS levels during osteoclastogenesis^43,44^. Most importantly, ROS production can increase Ca^2+^ signaling and activate NFATc1^45^. Furthermore, C-PC has known to be a potent ROS scavenger^18^. In agreement with these observations, our results demonstrated the ability of C-PC to attenuate RANKL-induced generation of ROS. Therefore, the reduction of intracellular ROS levels caused by C-PC could account for the inhibited osteoclast differentiation. No suppressive effect of C-PC was observed on osteoblast differentiation or mineralization, indicating the specificity of this compound on osteoclastogenesis and resorption.

Accumulating evidence suggests that c-Fos and NFATc1 are essential regulators of RANKL-induced osteoclastogenesis^46–48^. c-Fos is a significant component of the AP-1 complex, and it was shown that c-Fos-deficient mice developed osteopetrosis due to impaired osteoclastogenesis^48,49^. That effect was ultimately rescued to normal by ectopic expression of c-Fos^49^. NFATc1, which is critical for osteoclastogenesis, is regulated by c-Fos. The binding of the AP-1 transcription factor, which contains c-Fos, with the NFATc1 promoter, induces NFATc1 expression during osteoclastogenesis^46–48^. In addition, Costunolide, a sesquiterpene lactone, has shown the ability to suppress the transcriptional activity of c-Fos caused by RANKL and, therefore, inhibited the consequent expression of NFATc1^50^. The absence of NFATc1 in RAW 264.7 cells completely abolished RANKL osteoclastogenesis^51^. Also, mice deficient in NFATc1 exhibited defects in osteoclast differentiation and demonstrated symptoms of osteopetrosis^52^. Similarly, the inhibition of NFATc1 transcriptional activity attenuated RANKL osteoclastogenesis^53,54^. In the present study, we showed that C-PC significantly inhibited RANKL-induced c-Fos expression at the protein level. Consequently, RANKL-induced NFATc1 expression is also suppressed.

The canonical NF-κB signaling pathway is vital for osteoclastogenesis in response to RANKL^52^. NF-κB is constitutively present in the cytoplasm as a heterodimer that maintained its resting state by binding to the inhibitory subunit IκB-α^55^. RANKL treatment can induce the disintegration of IκB-α and therefore allows NF-κB unit to translocate into the nucleus and activates transcription^56^. Moreover, NF-κB can act as an upstream factor and mediate RANKL-induced c-Fos and NFATc1 expression^57^. In line with these observations, our study showed that C-PC treatment prevents the RANKL-induced degradation of IκB-α at all time points tested, indicating the ability of C-PC to counteract the RANKL activation of NF-κB, c-Fos, and NFATc1. Moreover, it is essential to mention that NF-κB was shown to control the early phase of osteoclast differentiation^57^. Therefore, we believe that early treatment of C-PC suppressed osteoclastogenesis by inhibiting NF-κB related pathway, while the late treatment does not affect. The limitations of the studies shown here are that the actual target site for ROS production is not revealed and also the mechanism by which C-PC acts as an inhibitor of the NF-κB pathway, which involves NFATc1 and c-Fos. Future studies will focus on these aspects.

The study in this report focuses on the effect of C-PC on RANKL-induced osteoclast differentiation (Fig. 6; schematic diagram). C-PC effect on differentiation was primarily measured by TRAP staining. A decrease in the expression of osteoclasts-specific markers such as *cathepsin K* and *integrin β3* corresponds with the reduced number of TRAP^+ve^ osteoclasts. C-PC significantly reduces RANKL-induced ROS generation. The underlying molecular mechanism of C-PC effect involves the inhibition of RANKL- induced NF-κB activation, which consequently reflects on the induction of c-Fos and NFATc1 (Fig. 6; schematic diagram). Our results clearly show that C-PC has inhibitory effects on RANKL-induced osteoclastogenesis via the suppression of NFATc1 and c-Fos activation. Hence, we suggest that C-PC could be a therapeutic candidate for the treatment of bone loss observed in conditions that demonstrate osteoclast activation (E.g., osteoporosis, rheumatoid arthritis, and periodontitis). The significance of this compound is that it does not affect osteoblast differentiation or activity.

## Methods

### Purchases and reagents

The RAW 264.7 cell line was purchased from American Type Culture Collection (ATCC® TIB-71™). C-PC was bought from Sigma-Aldrich (St. Louis, MO), dissolved in sterile water and stored at 4 °C. MTT assay kit, Alkaline phosphatase staining kit, and GAPDH antibody were also purchased from Sigma-Aldrich (St. Louis, MO). The following antibodies were bought from the company indicated in parenthesis: NFATc1 (SC-16657; Santa Cruz Biotechnology; Santa Cruz, CA), TRAP and CTSK (ab191406, ab19027; Abcam; Cambridge, United Kingdome), β3 Integrin, Caspase-3, caspase-9, c-Fos, and IκB-α (4702, 9662, 9504 S, 4384 S, 4814 S; Cell Signaling Technology; Danvers, MA), and HRP conjugated (mouse or rabbit) secondary antibodies (Santa Cruz Biotechnology; Santa Cruz, CA). Protein estimation reagents, molecular weight standards for proteins, and PAGE reagents were bought from Bio-Rad. Alizarin red solution was bought from Life-line Cell Technology (CM-0058; Fredrick, MD). Super Signal™ West Pico Chemiluminescent substrate was bought from Thermo Fisher Scientific (Waltham, Massachusetts**)**. Rhodamine phalloidin and other chemicals were purchased from Sigma-Aldrich (St. Louis, MO).

### Studies on osteoclasts

#### Preparation of osteoclast precursors from RAW 264.7 macrophage-like cell line

Murine osteoclasts were generated from RAW 264.7 cells as described^58^. Briefly, RAW 264.7 cells were plated at a low density in the presence of Dulbecco’s modified Eagle’s medium (DMEM) with 10% fetal bovine serum. After 24 h, the media was changed to α-MEM containing macrophage colony-stimulating factor (M-CSF; 10 ng/ml) and GST-RANKL (60 ng/ml) with or without the various concentration of C-PC (10,25, or 50 μg/ml). After two days, the medium was replaced with fresh M-CSF and RANKL with or without C-PC at indicated concentrations above. Recombinant GST-RANKL was purified as described previously^39^. Mature multinucleated osteoclasts were seen from day three onwards.

To further define the stage at which C-PC inhibits osteoclastogenesis, RAW cells were treated with C-PC at different stages. The treatment conditions are denoted as ‘*Early*, *Late*, *and Early/Late’* (illustrated in Fig. 1C). The treatment strategy is as follows: In the control treatment, RAW cells were treated with RANKL for 72 h. RANKL was added three times to RAW cell culture at 24 h interval (0 h, 24 h, and 48 h). Incubation was continued for 72 h, and osteoclasts were seen at ~72 h after treatment with RANKL. In the ‘*early’* treatment, RANKL and C-PC were added to RAW cell culture at 0 h, and incubation was continued for 24 h. After 24 h, cells were washed with cold PBS, and medium with RANKL was added at 24 h and 48 h intervals with no C-PC. Incubation was continued for 72 h. In the ‘*late*’ treatment, RANKL was added to RAW cells at 0 h, and 24 h, and the incubations were continued for 48 h. At 48 h, cells were washed, and the medium containing both RANKL and C-PC was added, and incubation continued for 72 h. In the ‘*Early/Late treatment’* strategy, the addition of both RANKL and C-PC was done at 0 h, 24 h, and 48 h. RANKL and C-PC were present in the culture for 72 h.

#### Tartrate-resistant acid phosphatase (TRAP)-staining

RAW 264.7 cells were used to generate osteoclasts as indicated above, in the presence or absence of various concentrations of C-PC. Undifferentiated macrophages were gently removed with cell stripper solution, and multinucleated cells were stained for TRAP. Briefly, cells were fixed with 4% paraformaldehyde, and then washed three times with Phosphate-Buffered Saline (PBS). TRAP staining was done with Leukocyte Acid Phosphatase Kit (Sigma; 387-A) according to the protocol provided by the manufacturer. Stained cells were photographed with phase-contrast microscopy, and images were processed in Adobe Photoshop (Adobe Systems Inc.).

#### MTT assay

MTT colorimetric assay analyzes the number of viable cells by the cleavage of tetrazolium salts added to the culture medium. MSM toxicity was assayed by measuring blue formazan formed from the 3-(4-5-dimethlthiazol-2-yl) 2-5-diphenyl tetrazolium bromide (MTT) salt by the cleavage of mitochondrial dehydrogenase enzyme (Sigma) as described previously^59^. RAW 264.7 cells were seeded at a density of 10000 cells/well in a 24-well flat-bottomed microtiter plate one day before the application of any treatment. Cells were incubated for 48 hours at various concentrations of C-PC. MTT was added to each well and incubated for 4 h at 37 °C. MTT solubilization solution provided by the manufacturer was added to the wells to stop the chemical reaction, and the plate was read at 570 nm as per instructions provided by the manufacturer (Sigma). Three to four wells were used for each treatment. Statistical significance was measured as described below.

#### Analysis of DAPI staining

RAW 264.7 cells were seeded on a 6-well plate at a density of 0.07 × 10^6^ and left overnight to attach. After 24 hours, cells were treated with C-PC (0, 10, 25, or 50 μg) for 48 hours. The cells were washed three times with PBS and then treated for 15 min with Triton X-100 to disrupt the cell membrane integrity. Nuclei were stained with a mounting solution containing DAPI (Vector Laboratories Inc., Burlingham, CA) at 37 C for 10 min in the dark. The cell nuclei were observed and photographed using fluorescence microscopy.

#### Semi-quantitative polymerase chain reaction (RT-PCR) analysis

RAW 264.7 cells were seeded at a density of 5 × 10^4^ cells/well in a 6-well plate. Cells were treated with RANKL and M-CSF in the presence or absence of various concentrations of C-PC for several time points as indicated in the study. Cells treated with only RANKL and M-CSF were used as controls. Qiagen RNeasy mini kit was used to extract RNA according to the manufacturer protocol, and cDNA was synthesized using the SuperScript ® III First-strand Synthesis System (Invitrogen, Carlsbad, CA) with two µg of total RNA. To determine the mRNA levels of β3 integrin, CTSK, TRAP, and GAPDH, we used the following steps for PCR reaction^60,61^: 5 minutes at 95 °C, 30 cycles of (30 s, 94 °C; 30 s, 58 °C; 30 s, 72 °C), 5 min at 72 °C. For c-Fos and NFATc1^62^, we used the following steps for PCR reaction: 32 cycles of (1 min, 94 °C; 1 min, 58 °C; 1 min, 72 °C). After amplification, the PCR products were separated by electrophoresis on a 2% agarose gel, stained with GelGreen ™, and visualized by G-box^63,64^. Primers are shown in Table 1.

**Table 1:** Primers used for the RT-PCR.

Gene	Forward primer	Reverse primer	~Amplicon length
β3 integrin	CCTTTGCCCAGCCTTCCA	GTCCCCACAGTTACATTG	305
CTSK	TTAATTTGGGAGAAAAACCT	AGCCGCCTCCACAGCCATAAT	400
TRAP	AGCAGCCAAGGAGGACTACGTT	TCGTTGATGTCGCACAGAGG	220
GAPDH	CCCACTAACATCAAATGGGG	ATCCACAGTCTTCTGGGTGG	324
c-Fos	ATGGGCTCTCCTGTCAACAC	GGCTGCCAAAATAAACTCCA	350
NFATc1	TGCTCCTCCTCCTGCTGCTC	CGTCTTCCACCTCCACGTCG	350

#### Rhodamine-phalloidine staining of actin filaments in osteoclasts

Osteoclasts were generated from RAW 264.7 macrophage-like cell line as described above. For staining, RAW 264.7 cells were plated on glass coverslips, and differentiation was done as described above with M-CSF and RANKL with and without 50 μg/ml C-PC. Mature multinucleated osteoclasts were seen from day three onwards. Undifferentiated RAW cells were gently removed with cell stripper solution (Sigma), and actin staining was performed as previously described^65,66^. Briefly, cells were rinsed with PBS containing 5 mM EGTA (PBS-EGTA) and fixed in 4% (w/v) paraformaldehyde in PBS-EGTA for 20 min at 37 °C. Coverslips were immersed in 47.5% ethanol containing 5 mM EGTA for 15 min at room temperature and rinsed with several changes of PBS-EGTA before staining with 1:100 dilution of rhodamine-phalloidin in PBS-EGTA for overnight at 4 °C. After rinsing several times with PBS-EGTA, coverslips were mounted on a mounting solution (Vector Laboratories Inc., Burlingham, CA). Actin stained osteoclasts were photographed with a Bio-Rad confocal lasers-scanning microscope. Images were stored in TIF image format and processed by Adobe Photoshop (Adobe Systems Inc., Mountain View, CA).

#### Resorption assay

Terminally differentiated osteoclasts were collected and replated on dentin slices for 72 hours. Cells were treated twice with RANKL in the presence or absence of 50 μg/ml C-PC. Resorption assay was performed as described previously^6,28^.

#### ROS

The DCFDA detection assay kit (2′,7′–dichlorofluorescein diacetate, also known as DCFH- DA), was used to detect intracellular ROS levels. ROS measurement assay was done as described^35^. Briefly, RAW 264.7 cells were treated with RANKL, M-CSF, and C-PC (25 or 50 μg) for 72 h. Intracellular ROS levels were determined using 2′,7′–dichlorofluorescein diacetate, which oxidizes into fluorescent DCF in the presence of ROS. Cells were washed in a buffer provided with the kit and incubated in the dark for 45 min with 10 μM DCFH-DA. Images were obtained using a fluorescence microscope.

#### Immunoblotting analysis

RAW 264.7 cells were seeded at a density of 5 × 10^4^ cells/well in a 6-well plate. Cells were treated with RANKL and M-CSF in the presence or absence of various concentrations of C-PC for several time points, as indicated in the study. Cells treated with only RANKL and M-CSF were used as controls. The cells were lysed with 1X Radioimmunoprecipitation assay buffer (RIPA) with a protease inhibitor and scraped with a cell scraper. Lysates were kept on the ice for 15 minutes and then centrifuged at 15,000 rpm for 15 minutes at 4 °C. The supernatant was collected, and the protein concentration was determined using Bradford assay. An equal amount of lysate proteins were used for analyzed by SDS-PAGE (10% gel) and transferred to a polyvinylidene fluoride (PVDF) microporous membrane. Membranes were blocked for 2 hours in 5% bovine serum albumin (BSA) in PBS with tween-20 (PBS-T) and incubated with the primary antibody of interest in PBS-T at the recommended dilution by the manufactures at 4 °C for overnight (ON). Membranes were washed three times with PBS-T and then incubated with species-specific HRP-conjugated secondary antibody in PBS-T at the recommended dilution by the manufactures at RT for 1 h. Immunoblotting with a GAPDH antibody (1:5000 dilution in PBS-T) was used as a loading control. After three washes for 5–10 min each, protein bands were visualized by chemiluminescence, an ECL kit^67,68^.

### Studies on osteoblasts

#### Alizarin red staining

Alizarin Red staining staining was done as described previously^69^. Briefly, UMR106 cells were seeded at a density of 4 × 10^5^ cells/well in a 6-well plate with and without C-PC (50 μg/ml) and incubated for 7 days. Cells grown in the osteogenic medium were used as a positive control. Cells were washed with PBS three times and fixed with absolute ethanol for 30 min at room temperature to evaluate the effect of C-PC on matrix mineralization. After aspiration of ethanol, 2% Alizarin red stain solution was added to each well until the cells were covered completely and incubated at room temperature for 45 min in the dark. Subsequently, wells were washed with deionized water three times to remove unincorporated excess dye. The plates were then scanned with the EPSON Perfection V200 Photo scanner. Magnified pictures of the wells were taken using phase-contrast microscopy and images were captured using a Nikon digital camera using 10X objective.

#### Alkaline phosphatase (ALP) activity assay

ALP activity assay was performed according to the manufacturer instructions and, as previously described^69^. Briefly, cells were seeded at a density of 4 × 10^5^ cells/well in a 6-well plate in the presence or absence of C-PC (50 μg/ml) for 7 days. Cells were washed with cold PBS three times and lysed with lysis buffer (50 mM Tris, 0.1% Triton-X100, 1 mM MgCl_2_, 100 mM glycine). Lysates were centrifuged at 14,000 RPM for 5 min. An equal amount of supernatant protein was used as triplicates in a 96-well plate to measure the activity. P-Nitrophenyl phosphate (10 µl; Sigma) was added to each well, and the absorbance was measured at 405 nm using a microplate reader (Cytation3 image reader) with software (Gen5 version 2.09).

#### Statistical analysis

Quantitative data are all expressed as mean ± SD, and statistical significance was determined using one - way ANOVA or Student T-test when applicable (Graph Pad Inc, San Diego, CA). The level of significance was set at P < 0.05.

## Supplementary information

Supplementary information.

## Data Availability

All data generated or analyzed during this study are included in this published article and its Supplementary Information files.
