# Supplementary material for: C-phycocyanin attenuates RANKL-induced osteoclastogenesis and bone resorption in vitro through inhibiting ROS levels, NFATc1 and NF-κB activation
Source: Sci Rep. 2020 Feb 13;10:2513. doi: 10.1038/s41598-020-59363-y (PMC7018981; doi:10.1038/s41598-020-59363-y)
Supplement: Supplementary file 1 — Supplementary information. [file 41598_2020_59363_MOESM1_ESM.docx]

**C-phycocyanin attenuates RANKL-induced osteoclastogenesis and bone resorption in vitro through inhibiting ROS levels, NFATc1 and NF-κB activation**

Mohammed S. AlQranei ^a,b^ Hanan Aljohani ^a,c^, Sunipa Majumdar ^a^, Linda T. Senbanjo ^a^ , and Meenakshi A. Chellaiah^a *^

^a^ Department of Oncology and Diagnostic Sciences, School of Dentistry, University of Maryland, Baltimore, MD, USA

^b^ Preventive Dental Sciences Department, School of Dentistry, Imam Abdulrahman Bin Faisal University, Dammam, Saudi Arabia

^c^ Department of Oral Medicine and Diagnostics Sciences, King Saud University, School of Dentistry, Riyadh, KSA

^*^**Correspondence:** Meenakshi Chellaiah

Department of Oncology and Diagnostic Sciences, School of Dentistry, University of Maryland, 650 W Baltimore Street, Baltimore, MD 21201,USA.

e-mail: mchellaiah@umaryland.edu . Tel: +1 (410) 706-2083.

**ORCID**

Meenakshi A. Chellaiah [*https://orcid.org/0000-0001-6572-1106*](https://orcid.org/0000-0001-6572-1106)

**Supplementary Figure S1: Analysis of cytotoxicity and cell viability by MTT assay.**

RAW cells were treated with varying doses of C-PC (0, 10, 25, 50, 150 μg/ml) for 48 hours. Cells were subjected to the calorimetric MTT assay, as described in the Materials and methods section, to determine the viability. Statistical analysis of the viability is provided as a graph. The viability was not affected at 10-100μg/ml. A significant decrease in the viability was observed at 150 μg/ml C-PC (*** p<0.001; n=3) as compared with untreated RAW cells. Experiment was repeated three times.


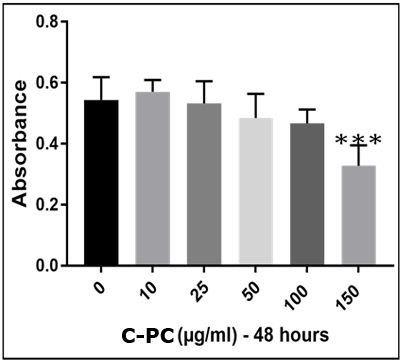


**Supplementary Figure S2. C-PC interfered with neither osteoblast differentiation nor mineralization activity.** UMR106 cells were stimulated with osteogenic media (OM) with the presence or absence of 50 μg C-PC for 7 days. **(A)** ALP activity for both groups was measured. **(B)** Alizarin Red Staining was performed to evaluate the effect of C-PC on the mineralization activity of osteoblast. * P < 0.05, t-test was applied**.**


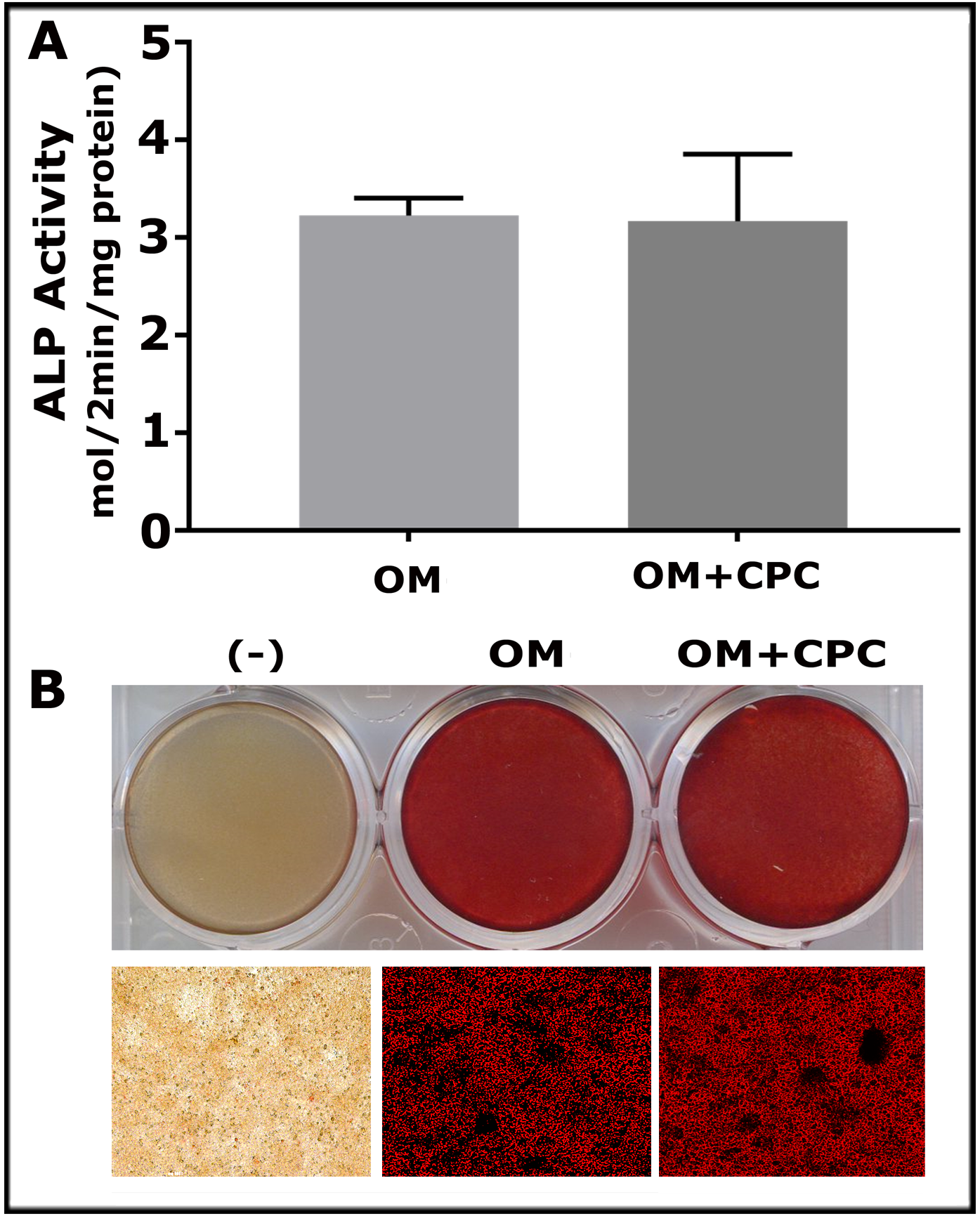

**Supplementary Figure S3. Analysis of the effect of C-PC on the expression of apoptotic markers.** Uncropped raw data for immunoblotting analyses shown in Figure 2A (lanes 1- 4) are provided. Red rectangle indicates the proteins which are shown in Figure 2A. The other lanes which were not marked by a rectangle in each autoradiogram represent different treatments which are not pertinent to the present studies.

**Supplementary Figure S4. Analysis of the effect of C-PC on the expression of osteoclast-specific markers.** Uncropped raw data for immunoblotting analyses shown in Figure 2C (lanes 4-6) are provided. Immunoblotting analysis for cathepsin K demonstrated multiple bands ( ~ 29, 37, and 42 kDa) which may represent different forms of the cathepsin K (CTSK). CTSK also contains a glycosylation site. Therefore, it can be seen at MW between 25-47 kDa. The ~29kDa protein could be a cleaved form of CTSK enzyme^1,2^. All of these bands show a similar pattern of a gradual decrease in the level in response to C-PC. We chose the ~29kDa protein band which represents the mature monomeric CTSK. Red rectangle in each autoradiogram indicates the proteins which are shown in Figure 2C. The other three lanes in each autoradiogram represent different treatments which are not relevant to the present studies.


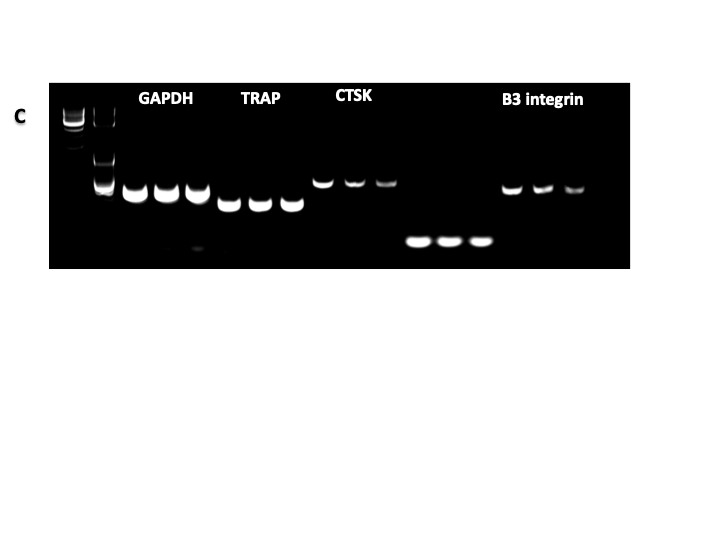


**Supplementary Figure S5. Analysis of the effect of C-PC on the expression of osteoclast-specific markers.** Uncropped raw data for RT-PCR analyses shown in Figure 2C (lanes 1-3) are provided. Only the indicated bands were used in Figure 2C.


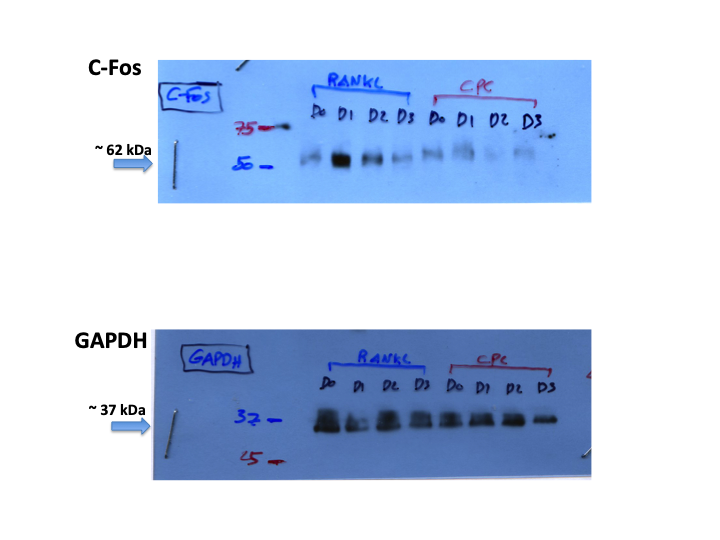


**Supplementary Figure S6. Immunoblotting analysis of the effect of C-PC on the expression levels of c-Fos.** Uncropped raw data are provided for data shown in Figure 4A.


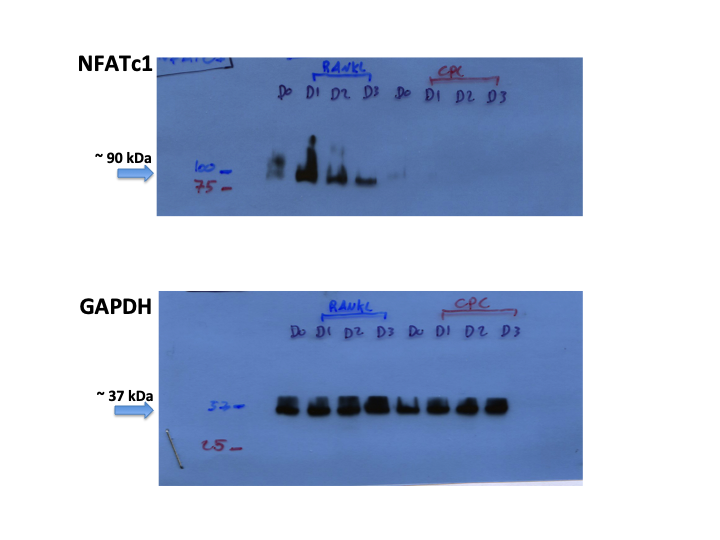


**Supplementary Figure S7. Immunoblotting analysis of the effect of C-PC on the expression levels of NFATc1.** Uncropped raw data are provided for data shown in Figure 4B.


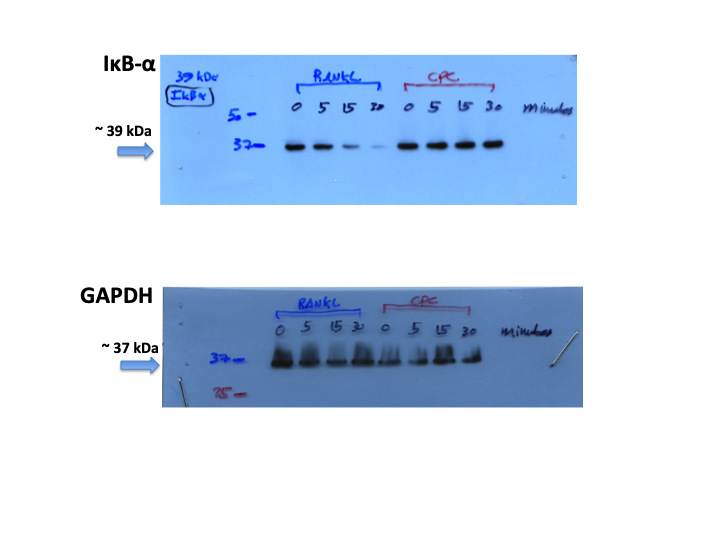


**Supplementary Figure S8. Immunoblotting analysis of the effect of C-PC on the expression levels of IκB-α.**  Uncropped raw data are provided for data shown in Figure 4C.


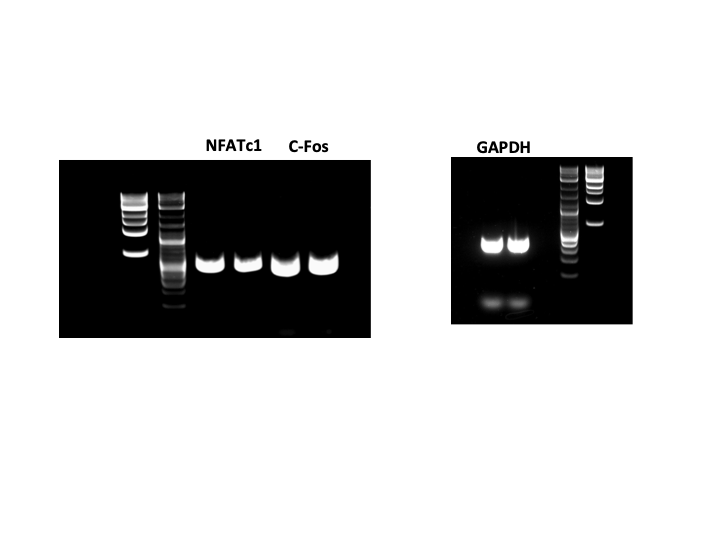


**Supplementary Figure S9. RT-PCR analysis of the effect of C-PC on the expression levels of c-Fos and NFATc1.** Uncropped raw data are provided for data shown in Figure 4D.


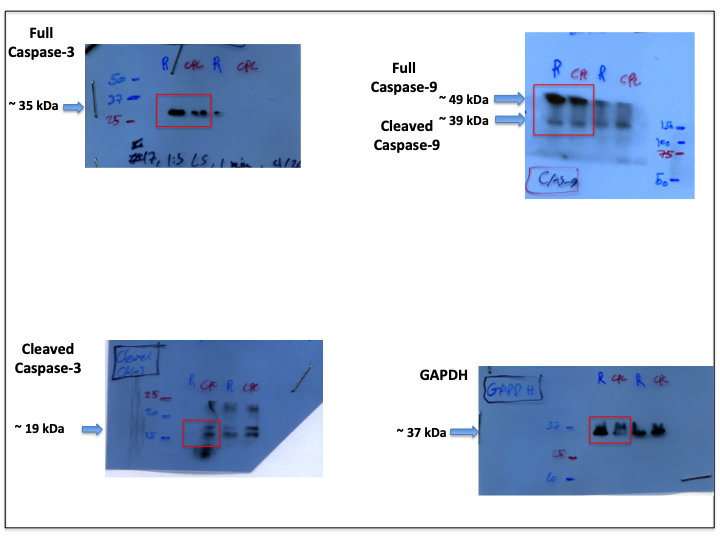


**Supplementary Figure S10. Apoptotic effects of C-PC on mature osteoclasts. (A)** Uncropped raw data provided for data shown in Figure 5D (Immunoblotting analysis). The other lanes which were not marked by a rectangle in each autoradiogram represent different treatments which are not pertinent to the present studies.

**References:**

1. Brömme, D., Okamoto, K., Wang, B. B. & Biroc, S. Human Cathepsin O2, a Matrix Protein-degrading Cysteine Protease Expressed in Osteoclasts. *J. Biol. Chem.* **271**, 2126–2132 (1996).

2. Hou, W. S. *et al.* Characterization of novel cathepsin K mutations in the pro and mature polypeptide regions causing pycnodysostosis. *J. Clin. Invest.* **103**, 731–8 (1999).
